# Dynamical interactions reconfigure the gradient of cortical timescales

**DOI:** 10.1162/netn_a_00270

**Published:** 2023-01-01

**Authors:** P. Sorrentino, G. Rabuffo, F. Baselice, E. Troisi Lopez, M. Liparoti, M. Quarantelli, G. Sorrentino, C. Bernard, V. Jirsa

**Affiliations:** Institut de Neurosciences des Systèmes, Aix-Marseille University, Marseille, France; Institute of Applied Sciences and Intelligent Systems, National Research Council, Pozzuoli, Italy; Department of Engineering, Parthenope University of Naples, Naples, Italy; Department of Motor Sciences and Wellness, Parthenope University of Naples, Naples, Italy; Institute for Diagnosis and Cure Hermitage Capodimonte, Naples, Italy; Biostructure and Bioimaging Institute, National Research Council, Naples, Italy

**Keywords:** Brain dynamics, Cortical gradients, Cortical timescales, Dynamical networks, Edge connectivity

## Abstract

The functional organization of the brain is usually presented with a back-to-front gradient of timescales, reflecting regional specialization with sensory areas (back) processing information faster than associative areas (front), which perform information integration. However, cognitive processes require not only local information processing but also coordinated activity across regions. Using magnetoencephalography recordings, we find that the functional connectivity at the edge level (between two regions) is also characterized by a back-to-front gradient of timescales following that of the regional gradient. Unexpectedly, we demonstrate a reverse front-to-back gradient when nonlocal interactions are prominent. Thus, the timescales are dynamic and can switch between back-to-front and front-to-back patterns.

## Author Summary

Cortical gradients have been proposed as a general gauge of brain interareal variability. In line with the expected sensory-to-associative hierarchical organization, brain regions fall, on average, along a back-to-front axis of variance of both structural and functional features. However, brain activity is dynamic and nonlinear, with large-scale interactions constantly reconfiguring over time. Analyzing a large cohort of magnetoencephalographic recordings in humans at the single-trial level, we discover a much richer repertoire of cortical timescales. Notably, a front-to-back gradient of timescales is observed when interactions across regions give rise to an emergent state of information exchange, which we uniquely capture by using edge-wise connectivity measures.

## INTRODUCTION

The human brain constantly scans the environment in search of relevant incoming stimuli, and appropriately reconfigures its large-scale activation according to environmental requests. The functional organization subtending these abilities, however, is not understood. One way to synthetically represent the functional organization among brain areas is by conceptualizing the brain as a network, whereby areas are represented as nodes, and structural connections or functional communication among them are represented as edges (Sporns, 2013). This approach led to the identification of a number of invariant features in the large-scale organization of the brain. From the functional point of view, large-scale patterns of interactions among areas are nonstationary and reorganize over time (Bassett et al., 2011; Deco et al., 2011; Pedersen et al., 2017; Sorrentino et al., 2021a). It has been hypothesized that healthy resting-state brain dynamics is tightly linked to the constant readiness to stimuli that vary across multiple timescales (Friston et al., 1995; McIntosh et al., 2008). Accordingly, the loss of efficient dynamics has been linked to brain disorders and clinical symptoms (Sorrentino et al., 2021a). Brain adaptability requires the quick acquisition of information from the environment, its integration and interpretation. Converging evidence suggests that a set of communication channels with intrinsic operational times is present, which results in spatiotemporally nested activities (Deco et al., 2009; Pillai & Jirsa, 2017). In this line of thinking, it has been shown in both human and macaque that, regardless of the presence of a stimulus, areas that are hierarchically lower in information processing operate at higher speed as compared to associative areas (which integrate information) (Gao et al., 2020; Müller et al., 2020; Murray et al., 2014, 2017; Shafiei et al., 2020). Hence, the cortex appears to be organized along a back-to-front gradient of timescales.

Such a gradient exclusively refers to local information processing at the regional level (network nodes). However, acquisition, integration and interpretation of inputs are distributed and dynamical processes, relying on the reconfiguring interactions (functional edges) occurring *between* regions. Hence, we hypothesized that the corresponding timescales should also be present between regions.

To test our hypothesis, we used source-reconstructed magnetoencephalographic (MEG) data from a cohort of 58 healthy young subjects, based on the Automated Anatomical Labeling (AAL) atlas, and we analyzed the time-resolved correlations between all pairs of brain regions, as a proxy of the dynamical interactions.

## RESULTS

**Figure 1.**
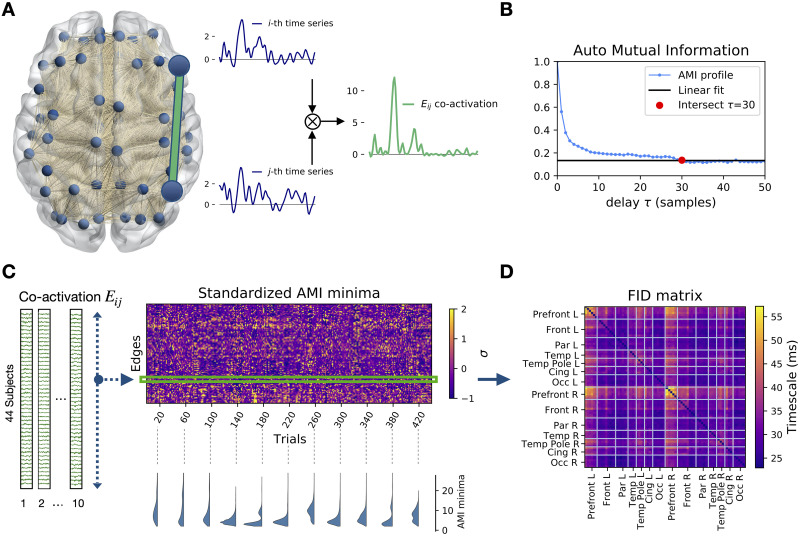
Auto-mutual information analysis of the edge time series in MEG data. (A) The preprocessed magnetoencephalography (MEG) dataset consists of *N* = 78 source-reconstructed signals, one for each brain region. We define the edge time series *E*_*ij*_(*t*) (green) as the element-wise product of nodal z-scored signals at *i* and *j* (blue). (B) Each point in the plot represents the mutual information between the co-activation time series and its *τ*-delayed copy. The red dot represents the delay at which the AMI reaches a minimal level (defined by a linear fit of the AMI tail). Notice that each time step *τ* corresponds to 3.9 ms, given the sample frequency of 256 Hz. (C) For each of the 44 subjects, the time series are split into ten, 10-s-long segments (epochs). For each trial, the *τ* corresponding to the AMI minima is computed. In the carpet plot, the minima are standardized across edges for each trial separately. The example minima distributions (bottom) in randomly selected epochs show that multimodality can emerge naturally (D) Averaging over the standardized AMI minima, we obtain the *N* × *N* FID matrix, where rows and columns are regions, and the matrix elements are the average minima across trials. For each couple of brain regions, this matrix describes the typical time that the information is preserved in the corresponding co-activation signal.

For each edge in the brain network we defined a time series as the co-activations of the MEG signals at the extremal nodes (Figure 1A) (see Materials and Methods). We used auto-mutual information (AMI) to measure the amount of statistical dependence between any co-activation time series and its time-delayed copy (MacKay, 2019). Repeating this operation for several delays, a profile of information decay was drawn for each edge of the brain network (Figure 1B). For short time delays, the high value of the AMI indicates little information loss. The AMI drops (loss of information) as the time delay increases. A fast/slow characteristic decay time indicates fast/slow information loss.

**Figure 2.**
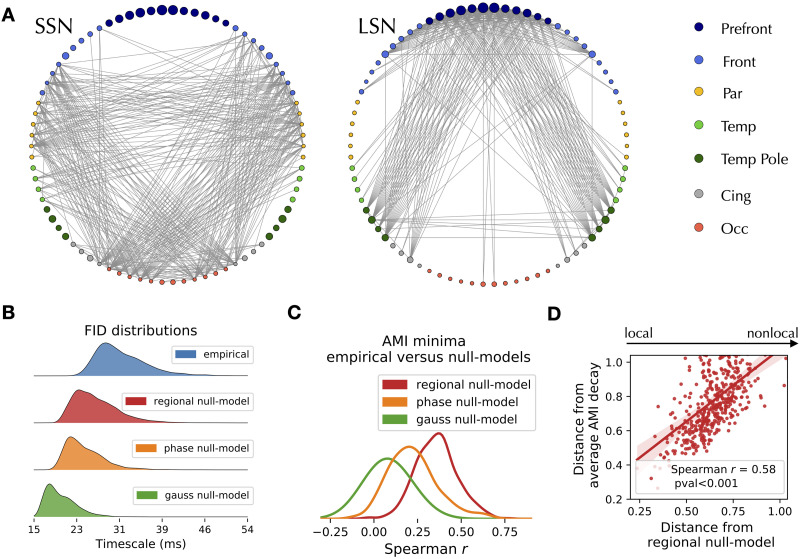
Topography of delays and surrogate analysis. (A) The edges with the fastest (left) and slowest (right) decay times split into a short-storage and a long-storage network (SSN and LSN) at the trial-averaged level. (B) Distributions of the average edge AMI decays in the original (blue), regional (red), phase (orange), and Gaussian (green) null models show that the long decay times of information depend on the dynamics of the edges and are not explained by nodal spectral features or by static correlations alone. (C) Distribution of the correlation between the decay times of each trial with the corresponding null models. (D) The *x*-axis measures the distance (1 minus the Spearman’s correlation) of each trial from the null model, which is used to represent the amount of nonlocal interactions in each trial. The *y*-axis represents the distance from the FID matrix (averaged across empirical trials). Each dot represents a trial.

AMI edge decay times are organized according to a characteristic spatiotemporal pattern (Figure 1C, top right). The trial-specific distributions of the decay times can be both multimodal and unimodal, showing that the brain can dynamically rearrange into subnetworks operating at different timescales (examples are shown in Figure 1C, bottom right). Averaging across trials, we define the functional information decay (FID) matrix (Figure 1D), which reveals a temporal hierarchy of the edges. Considering the edges with the lowest and highest retention of information in the FID matrix, we identify two subnetworks: the short- and long-term storage network (SSN, Figure 2A, *left*, and LSN Figure 2A, *right*, respectively). The SSN spans regions related to stimulus processing, while the LSN mainly involves regions related to higher cognitive functions (Table 1). We conclude that the hierarchy of timescales, consistent with the previously described nodal hierarchy, is manifest at the edge level (on average). Average information storage at the edge level ranges from 16 to 55 ms. However, at the single-trial level, we find a larger range, with decays varying between 3.9 to 277 ms.

**Table 1.**
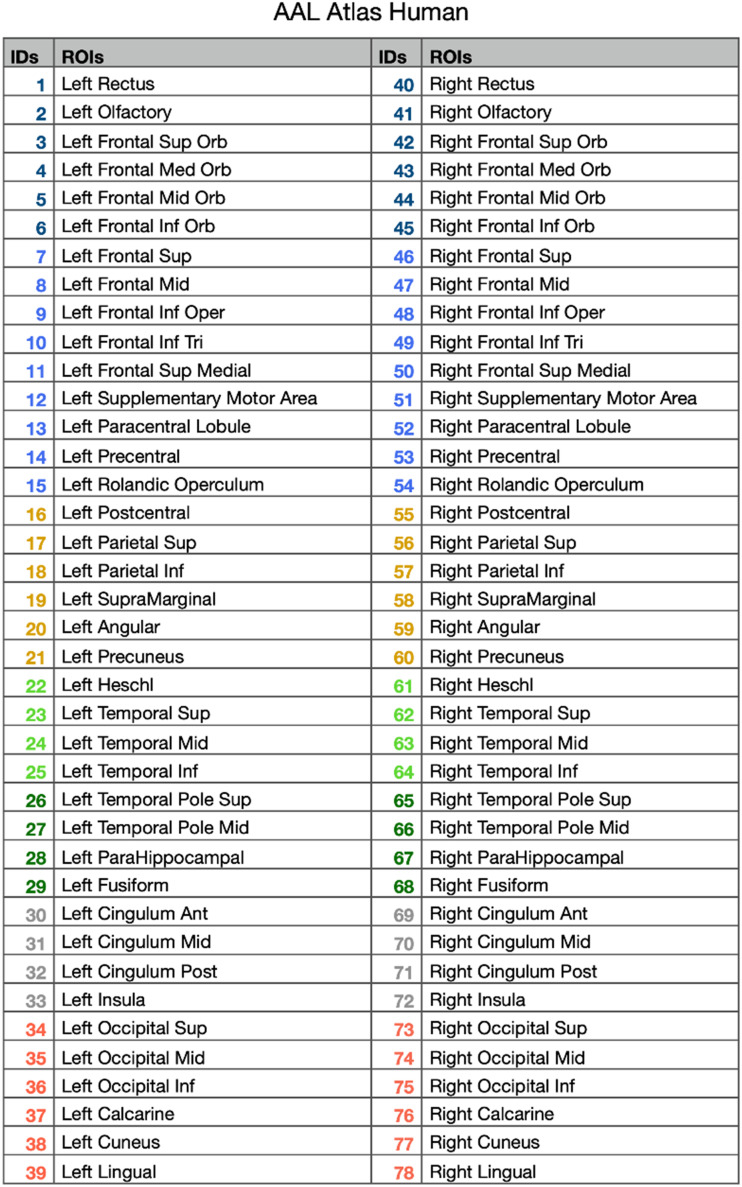
List of regions of AAL Atlas

*Note*. Each region number is colored according to the lobe. Color codes are reported in Figure 2A. ROIs = regions of interest.

The average edge decay times vary smoothly across the brain, spanning between the SSN and the LSN (Figure 2B, blue). Edge decay time may capture nonlocal interactions between regions or merely reflect local nodal processes. To distinguish between the two possibilities, we generate three null models to disrupt or disregard dynamic interactions between regions: (1) Gaussian surrogates, where the observed nodal autocorrelations (i.e., the power spectrum) are imposed on otherwise independent processes; (2) phase-randomized surrogates preserving both the regional autocorrelations and the pairwise static correlations (i.e., preserving static functional connectivity while disrupting the dynamics); and (3) regional null models, where the edge decay times are defined as the geometric mean of the AMI decay times estimated from nodal time series (*τ*_*ij*_ = $\sqrt{\tau_{i}\tau_{j}}$). None of the three null models reproduces the empirical decay-distribution (Figure 2B). We conclude that edge time delays characterize dynamical interactions between regions.

To test whether the topographical organization of the edge decay times emerges from local or distributed processes, we correlate the empiric AMI decays with the decays retrieved by the null models. At the trial-averaged level, the FID matrices derived from the null models correlate with the empirical ones (Spearman’s *r*_*s*_ = 0.89, *r*_*s*_ = 0.94, *r*_*s*_ = 0.95, for the Gaussian, phase-randomized, and regional null models, respectively, *p* < 0.001 for all cases). However, a greater variability of the correlations exists at the single-trial level (Figure 2C), with trials that do not show significant correlations with the corresponding null models. The regional null model is the closest one to the empirical data, thus we selected it for further analyses. Since the regional null model only retained local properties, we classified each empirical trial according to its distance from the corresponding null model (defined as 1 minus the Spearman’s correlation coefficient in Figure 2D). Hence, a trial that is distant from its null model is one that possesses prominent nonlocal features. Notably, the more an empirical trial has nonlocal interactions, the more it is distant from the empirical (average) FID matrix (*r*_*s*_ = 0.58; Figure 2D). To summarize, we have discovered a set of trials that possess significant nonlocal (edge) properties, which makes the topography of timescales deviate from the average configuration.

**Figure 3.**
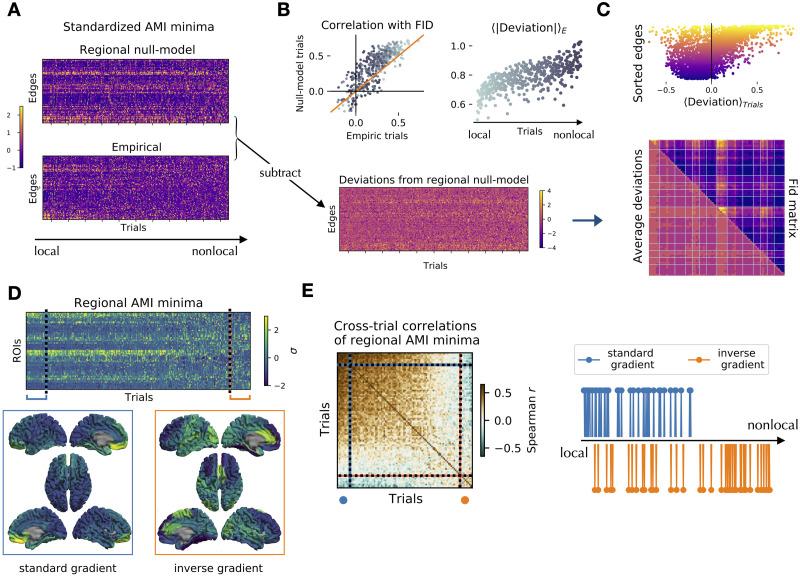
Trial-specific topography of edge and nodal timescales. (A) Standardized edge decay times (AMI minima) of the regional null model (top) and the empirical trials (bottom). The trials (matrix columns) are sorted according to a local-to-nonlocal axis, with the highest (lowest) correlation between the empirical and null model trials in the first (last) columns. (B) Bottom: the empirical minima (panel A, bottom) are subtracted from the null model minima (panel A, top) to define the trial-specific deviations from the null model. Top left: correlation among the FID matrix (trial average of the empirical AMI minima) and the trial-specific AMI minima in the empirical (*x*-axis) and null model (*y*-axis). Most trials are above the diagonal (orange), showing that the null model retrieves the empirical average topography of timescales better than the empirical single trials. Dots represent local and nonlocal trials from light to dark gray. Top right: the average edge deviations (in absolute value) progressively increase when nonlocal interactions take place. (C) While the decays of the SSN edges (purple) are consistently slower than the FID matrix (top, negative deviation), the LSN edges (yellow) vary more, and both slower and faster decays are observed (widespread deviations along the *x*-axis). Overall, the pattern of deviations across trials (bottom, lower triangular matrix) has a similar topography to the average FID matrix (bottom, upper triangular matrix) with Spearman’s *r*_*s*_ = 0.79. (D) Standardized nodal decay times (AMI minima) of empiric trials sorted by their similarity to the trial average (top). The trials with highest similarity show the known back-to-front gradient of timescales (bottom, left). The furthest trials from the mean display an inverse gradient (bottom, right). (E) Correlation matrix of the regional AMI minima (for the nodal timescales) between all pairs of trials. The trials are sorted as in panel D. The correlation matrix displays two major blocks corresponding to the standard and inverse-gradient trials. The standard-gradient trials correspond to the trials defined as local based on the edge decay times (the strongest 10% of these are represented as blue dots in the right panel). The inverse-gradient trials (orange dots, representing approximately 10% of the total) are predominantly nonlocal.

Strikingly, the corresponding trial in the null model recovers the empirical average topography (Figure 3A; delays are standardized along the edges within each trial). In fact, the correlation between the empirical trial and the empirical FID matrix is lower than the correlation between the corresponding surrogate trial and the empirical FID matrix (Figure 3B, top left. Note that most correlations fall above the diagonal, represented by the orange line. Local-to-nonlocal trials are represented in colors from dark to light gray). Subtracting the standardized empirical delays from the null model ones (Figure 3B, bottom), we show that the magnitude of the deviation from the null model is higher for nonlocal trials (Figure 3B, top right). Averaging the deviations across trials, we show high correlation between the average FID matrix and the average deviation matrix (Figure 3C, bottom; *r*_*s*_ = 0.79). That is, the edges manifesting faster dynamics than expected from the regional null model (negative average deviations) are roughly corresponding to the LSN, while slower than expected edges (positive average deviations) generally belong to the SSN (Figure 3C, top; edges are sorted according to trial-average delay. Color map as in Figure 1D). We note that LSN (Figure 3C, top; yellow) edges are characterized by higher deviation variability as compared to the SSN ones (Figure 3C, top; purple).

We then explore the relationship between nonlocal interactions and the known nodal gradient of timescales (Gao et al., 2020; Kiebel et al., 2008; Murray et al., 2014). Computing the AMI decay times of the nodal time series and sorting trials in growing order of similarity with the trial-average pattern, we recover the well-established back-to-front gradient of timescales (Figure 3D, bottom left). Remarkably, the trials that are the least similar to the average reveal an inverse front-to-back gradient (Figure 3D, bottom right). The matrix of cross-trials correlations (Figure 3E, left) shows that standard- and inverse-gradient trials are similar to themselves and anticorrelated to each other. Finally, most of the trials with an inverse front-to-back gradient are dominated by nonlocal higher order interactions between regions (Figure 3E, right; Spearman’s *r*_*s*_ = 0.42 between the degree of nonlocality as observed from edge analysis and the distance from the average nodal gradient). All the results of the manuscript were replicated on the Desikan-Killiany-Tourville (DKT) atlas and selecting different epochs for each subject (see Supporting Information Figure S4). Furthermore, the results could not be replicated based on random surrogates that had been linearly mixed according to subject-specific lead field matrices (see Supporting Information Figure S1). This makes it unlikely that our results could be explained by volume conduction alone. However, the reader should keep in mind that source-reconstruction is a complex, ill-posed problem and, as such, some uncertainty remains for the source estimates.

## DISCUSSION

Cortical gradients provide an organizational framework for capturing the topography of large-scale cortical organization, identifying the major axes of variance in cortical features such as gene expression, cell densities, axonal connectivity (Fulcher et al., 2019), and neuronal timescales (Kiebel et al., 2008), among others. However, brain activity is ever changing and the topography of functional features is expected to vary accordingly. Analyses of functional gradients based on trial average might hide this variability. Furthermore, the classical focus on the nodal features, that is, on region-specific signals, could disregard the impact of potential emergent phenomena on the gradient flexibility.

Utilizing edge-wise analyses, our results reveal that nonlocal interactions play a key role in shaping whole-brain activity and are associated with variability in the cortical gradients of timescales. In fact, in addition to the classical back-to-front gradient, which mostly reflects local information processing, nonlocal interactions were associated with an inverse front-to-back gradient. We propose that interactions between brain regions encode information beyond the purely nodal activity, and that such information is retained according to edge-specific characteristic lifetimes. Hence, edge-wise analyses can be seen as complementary to node-wise analyses. However, to correctly interpret the results of the edge time series as a genuine manifestation of nonlocal interactions, it is important to demonstrate that the results cannot be derived from static nodal properties alone (Novelli & Razi, 2022). To this end, we tested our results against three null models, each preserving nodal and/or static correlation features, while disrupting the temporal structure of the interactions. The surrogates were not always capable of conveying all the information contained in the edge time series. This allowed us to distinguish moments when nonlocal interactions were occurring, from moments where they were not. Hence, we could classify the trials on an axis of increasing “nonlocality,” according to the distance from the null models (Figure 3). On the one hand, when the edge time series did not beat the nulls (i.e., the observations were entirely explained by nodal null models), the standard back-to-front gradient was observed. On the other hand, if nonlocal interactions were present (i.e., edge time series beat the nulls), the gradient rearranged itself across the cortex (Figure 3B–D). Remarkably, in a number of trials, the topography of timescales anticorrelated with the standard gradient (Figure 3E), giving rise to an inverse front-to-back gradient.

The presence of nonlocal interactions in a subset of trials can be interpreted within the integration/segregation hypothesis (Shine et al., 2016), which suggests that the brain alternates moments in which the processing of information is local to moments of collective processing. If this is the case, our technique can be used as an alternative measure of network integration (Wang et al., 2021). Furthermore, we suggest that these moments of emergent nonlocal interactions along the edges might be related to increased presence of traveling waves (Roberts et al., 2019) and/or large-scale bursts of activity, such as neuronal avalanches (Shriki et al., 2013; Sorrentino et al., 2021b). The distribution of the average information decays revealed two subnetworks with short (SSN) and long (LSN) storage capability. This result is in line with multiple models (Engel et al., 2001) and experimental evidence (Buschman & Miller, 2007) showing that processing of external stimuli involves (bottom-up) perception and abstraction, as well as (top-down) interpretation according to expectations (priors), embodied in the internal brain state (Engel et al., 2001). For further considerations on the neurophysiological underpinnings of the SSN and LSN networks, please refer to the Supporting Information section *Detailed analysis of the average edge topography*. Interestingly, single-trial analysis of the decay times revealed a spontaneous and dynamic clusterization of timescales (i.e., multimodality; Figure 1C), which lends itself for further topographical characterization in future studies.

In conclusion, the dynamic rearrangement of cortical gradients speaks to a dialectic interaction between top-down and bottom-up processes, which remains open to interpretation while stressing once again the relevance of distributed dynamic brain processes underpinning cognition.

## MATERIALS AND METHODS

### Participants

Fifty-eight right-handed and native Italian speakers were considered for the analysis. To be included in this study, all participants had to satisfy the following criteria: (1) to have no significant medical illnesses and not to abuse substances or use medication that could interfere with MEG/EEG signals; (2) to show no other major systemic, psychiatric, or neurological illnesses; and (3) to have no evidence of focal or diffuse brain damage at routine MRI. The study protocol was approved by the local Ethics Committee. All participants gave written informed consent.

### MRI Acquisition

Three-dimensional T1-weighted brain volumes were acquired at 1.5 Tesla (Signa, GE Healthcare) using a 3D magnetization-prepared gradient-echo BRAVO sequence (TR/TE/TI 8.2/3.1/450 ms, voxel 1 × 1 × 1 mm^3^, 50% partition overlap, 324 sagittal slices covering the whole brain).

### MEG Acquisition

Subjects underwent magnetoencephalographic examination in a 163-magnetometers MEG system placed in a magnetically shielded room (AtB Biomag UG, Ulm, Germany). The preprocessing was done similarly as in Sorrentino et al. (2018). In short, the position of four coils and of four reference points (nasion, right and left preauricular point and apex) were digitized before acquisition using Fastrak (Polhemus). The brain activity was recorded for 7 min, with eyes closed, with a ∼1.5-min-long break at 3.5 minutes, so as to minimize the chances of drowsiness. During the break, the patients were waiting inside the shielded room and they were informed that there was a pause, and that they were allowed to adjust their position if they felt the need to do so. Hence, the head position was recorded at the start of each segment. The data were sampled at 1024 Hz, and a fourth-order Butterworth band-pass filter was applied to select components between 0.5 and 48 Hz. During the acquisitions, electrocardiogram (ECG) and electrooculogram (EOG) were also recorded (Gross et al., 2013). These steps were done using Matlab 2019a and the Fieldtrip toolbox 2014 (Oostenveld et al., 2011).

### Preprocessing

Principal component analysis was performed to reduce the environmental noise (de Cheveigné & Simon, 2008; Sadasivan & Narayana Dutt, 1996). Noisy channels were removed manually through visual inspection of the whole dataset by an experienced rater. Supervised independent component analysis was performed to eliminate the ECG (and the EOG) component from the MEG signals (Barbati et al., 2004). Trials that did not contain artifacts (either system related or physiological) or excessive environmental noise were selected.

### Source Reconstruction

The data were coregistered to the native MRI. A modified spherical conductor model was used as a forward model (Nolte, 2003). The voxels in the MRI were labeled according to the AAL atlas (Tzourio-Mazoyer et al., 2002) and the DKT atlas (Alexander et al., 2019). We used the cortical regions for a total of 78 areas of interest (66 for the DKT). Subsequently, a linearly constrained minimum variance beamformer was used to compute 78 (for the AAL) time series (one per area of interest) at a sampling frequency of 1024 Hz (Van Veen et al., 1997). Reconstructed sources were again visually inspected by an experienced rater. Of the initial 58 subjects, 44 had enough artifact-free acquisitions and were selected for further analysis. The source-reconstructed signals were downsampled to 256 Hz.

### Edge-Centric Approach to MEG

In this work, we adopted an edge-centric approach that, rather than focusing on the local activity of the regions (nodes), represents the dynamics of the interactions between couples of brain regions (Esfahlani et al., 2020). This allowed us to characterize the whole-brain network activity in terms of dynamical nonlocal interactions, highlighting the relational properties of each couple of nodes. Given any couple of nodes *i* and *j* and their respective source-reconstructed signals *X*_*i*_(*t*) and *X*_*j*_(*t*), we defined a characteristic time series *E*_*ij*_ for the edge *ij* as the product of the z-scored signals, that is,

$$E_{ij}\left(t\right)=\frac{X_{i}\left(t\right)-{\overset{ˉ}{X}}_{i}}{\sigma \left(X_{i}\right)}\cdot \frac{X_{j}\left(t\right)-{\overset{ˉ}{X}}_{j}}{\sigma \left(X_{j}\right)},$$

where $\overset{ˉ}{X}$ and *σ*(*X*) denote the mean and variance of the signals, respectively. One can interpret the edge co-activation time series as the unfold in time of the pairwise correlations. In fact, the average of the above expression over time corresponds to the Pearson correlation between the signals at nodes *i* and *j*. The edge time series were further analyzed by information theoretic measures, aiming at characterizing the information storage capability of each functional edge.

### Estimation of Information Decay Time Through Mutual Information

Shannon Entropy, defined as

$$H\left(X\right)=-\sum_{x_{i}}\;P_{X}\left(x_{i}\right)\;\log\left(P_{X}\left(x_{i}\right)\right),$$

quantifies the uncertainty over the possible outcomes *x*_*i*_ of a random variable X with probability distribution *P*_*X*_. If the uncertainty over future outcomes of *X* decreases as we measure the outcome *y*_*i*_ of another random variable *Y*, we conclude that *X* and *Y* represent two processes that are not independent. The new resulting uncertainty over *X* is then defined by

$$H\left(X|Y=y_{i}\right)=-\sum_{x_{i}}\;P_{XY}\left(x_{i}|y_{i}\right)\log\left(P_{XY}\left(x_{i}|y_{i}\right)\right)=-\sum_{x_{i}}\;\frac{P_{XY}\left(x_{i},y_{i}\right)}{P_{Y}\left(y_{i}\right)}\log\left(\frac{P_{XY}\left(x_{i},y_{i}\right)}{P_{Y}\left(y_{i}\right)}\right),$$

with *P*_*XY*_(*x*_*i*_, *y*_*i*_) denoting the joint probability distribution of the pair (*X*, *Y*). The weighted sum over all possible outcomes of *Y* defines the conditional entropy, that is, the uncertainty of *X* conditioned on *Y*

$$H\left(X|Y\right)=-\sum_{y_{i}}P_{Y}\left(y_{i}\right)\;H\left(X|Y=y_{i}\right)=-\sum_{x_{i},y_{i}}\;P_{XY}\left(x_{i},y_{i}\right)\log\left(\frac{P_{XY}\left(x_{i},y_{i}\right)}{P_{Y}\left(y_{i}\right)}\right)=H\left(X,Y\right)-H\left(Y\right),$$

where

$$H\left(X,Y\right)\equiv \sum_{x_{i},y_{i}}\;P_{XY}\left(x_{i},y_{i}\right)\log\left(P_{XY}\left(x_{i},y_{i}\right)\right).$$

The reduction in uncertainty (or, equivalently, the increase of information) over *X* given by the knowledge of *Y* is measured by the mutual information (MI)

$$I\left(X,Y\right)=H\left(X\right)-H\left(X|Y\right)=H\left(X\right)+H\left(Y\right)-H\left(X,Y\right).$$

Unlike other measures, such as partial autocorrelation, MI statistical dependencies that take into account nonlinear interactions, which are ubiquitously found in brain data (Paluš, 1996; Stam, 2005). In order to quantify the time span before the information in a signal *X*(*t*) is lost, we rely on the AMI, that is, the MI between the signal and its time-delayed copy *Y* = *X*(*t* − *τ*). According to previous works on M/EEG (Gómez et al., 2007; Jeong et al., 2001), a stable estimate of the probability distribution of any real-valued signal *X*(*t*) is obtained by dividing the data into 64 bins. The joint probability distribution for the pair (*X*(*t*), *X*(*t* − *τ*)), needed for the evaluation of the AMI, is given by a 64 × 64 contingency matrix, which is the joint histogram of the two variables. The AMI decays as a function of the delay *τ*, from a maximal value at *τ* = 0 to a relatively stable lower value as *τ* → ∞. The more gentle (“slower”) the decay, the longer the information lasts in the signal. It should be mentioned that there exists no unique estimator for information storage. We chose AMI since we were interested in an interval estimate, rather than a point estimate (see, e.g., Wibral et al., 2014). The same algorithm was used to compute the nodal decay times based on the nodal time series.

### Information Storage Capability of the Functional Edges

For each co-activation signal *E*_*ij*_, we estimated the AMI profile (Figure 1B) and we evaluated the time delay *τ* corresponding to the AMI reaching a stable minimum, that is, when the original signal and its *τ*-delayed versions have become maximally independent. For all the edges, the decay times occurred within a maximum of 48 time steps (128 time steps in total). Therefore, in order to have a steady estimate of the baseline, we fitted the last 80 points of every AMI profile to a straight line, so as to find the stable minimum. Then, we found the *τ* corresponding to the moment where the AMI decay falls within a threshold, defined as 1 standard deviation from the stable minimum. Examples of the estimate of the AMI minimum for different edges, at the single-epoch level, are shown in Supporting Information Figure S2. An analysis for different thresholds (number of standard deviations around the stable minimum) is found in Supporting Information Figure S3. Averaging across all the trials (10 time windows of 10 s each across 44 subjects), we found the *N* × *N* FID matrix (where *N* = 78 is the number of brain regions), containing the decay times for each edge (Figure 1D). The AMI analysis of the coactivations shows that the decay times are different among edges, as revealed by the histogram in Figure 2B (blue). Selecting the edges from either the left or right tails of the distribution leads to the appearance of two topographically organized subnetworks (Figure 2A).

### Surrogate Analysis

#### Leakage analysis.

We designed surrogate analysis to exclude that linear mixing alone might spuriously generate the patterns observed in the FID matrix. To this end, we generated for each subject N Gaussian time series, with N = number of regions. Then, the subject-specific leadfield matrix was used to reconstruct the sensor signals for each subject. White noise correlated as 1/sensor distance, was added to the sensor time series with SNR = 12. Following this step, the sensor time series were inverted and new surrogate source-level time series were generated. On these source-level surrogates, we have computed the edge time series and the FID matrix as described previously.

#### Time-shuffled and phase-randomized surrogates.

First, we sought to investigate if the observed decay times might be derived by the spectral nodal properties alone. To this end, we generated N Gaussian processes, with N = number of regions, we fourier-transformed them, and we multiplied the resulting power spectra by the power spectra of the observed time series. Finally, we antitransformed and obtained surrogate time series that are independent Gaussian processes endowed with the same spectral power as the original data (Gaussian surrogates). Secondly, we sought to investigate if the time decays convey a dynamical feature of the time series or, alternatively, if they can be explained by static correlations. Hence, starting from the original data, we generated surrogates preserving not only the nodal spectral properties but also the cross-spectrum (static functional connectivity). To this end, we shifted by a random phase (extracted from a unimodal distribution) each frequency of the Fourier-transformed nodal signals. The same shift was uniformly applied to each region. Hence, we obtained new surrogates that preserve the functional connectivity while not showing the dynamic of the original data.

## ACKNOWLEDGMENTS

The authors thank Michele Allegra for insightful discussions.

## SUPPORTING INFORMATION

Supporting information for this article is available at https://doi.org/10.1162/netn_a_00270.

## AUTHOR CONTRIBUTIONS

Pierpaolo Sorrentino: Conceptualization; Data curation; Formal analysis; Funding acquisition; Investigation; Methodology; Supervision; Validation; Visualization; Writing – original draft; Writing – review & editing. Giovanni Rabuffo: Conceptualization; Formal analysis; Funding acquisition; Investigation; Methodology; Validation; Visualization; Writing – original draft; Writing – review & editing. Fabio Baselice: Formal analysis; Methodology; Writing – review & editing. Emahnuel Troisi Lopez: Data curation; Investigation; Methodology; Writing – review & editing. Marianna Liparoti: Methodology; Validation; Visualization. Mario Quarantelli: Validation; Writing – original draft. Giuseppe Sorrentino: Data curation; Funding acquisition; Investigation; Writing – original draft; Writing – review & editing. Christophe Bernard: Formal analysis; Methodology; Writing – review & editing. Viktor Jirsa: Formal analysis; Funding acquisition; Investigation; Methodology; Writing – original draft; Writing – review & editing.

## FUNDING INFORMATION

The work was supported by the University of Naples Parthenope “Ricerca locale” grant, by the grant ANR-17-CE37-0001-CONNECTOME, by the European Union’s Horizon 2020 Research and Innovation Programme under grant agreement No. 945539 (SGA3) Human Brain Project and VirtualBrainCloud No. 826421.

## TECHNICAL TERMS

Brain dynamics: The study of the temporal evolution of the brain activity, including the information flow across brain regions and the variation of their statistical dependences.
Co-activation time series: A time series obtained as the element-wise product of any pair of regional signals. Note that its time-average defines the Pearson’s correlation.
Auto-mutual information: Mutual Information between a signal and its time-delayed copy.
Magnetoencephalography: A device that measures the very small magnetic fields induced by neuronal activity.
Information storage: A measure of how long information is retained by a signal. It is defined as the shortest time-delay that minimizes the Auto-Mutual Information of a signal. This measure defines a characteristic timescale for the signal.
Surrogates: Obtained from the original data by destroying specific feature. If a measure of interest cannot be obtained from the surrogate data, then the destroyed feature is deemed necessary for the measure of interest to occur.
Source-reconstruction: A procedure to estimate the activity at a given location that has generated the signals recorded by the sensors.
Cortical gradient: A continuous axis of variance of a brain feature across the cortex.
Mutual information: Reduction in uncertainty about one observed signal given the knowledge of another reference signal.
