# Supplementary material for: Dynamical interactions reconfigure the gradient of cortical timescales
Source: Netw Neurosci. 2023 Jan 1;7(1):73–85. doi: 10.1162/netn_a_00270 (PMC10270712; doi:10.1162/netn_a_00270)
Supplement: Supplementary file 1 [file netn-7-1-73-s001.pdf]

Sorrentino, P, Rabuffo, G., Baselice, F., Troisi Lopez, E., Liparoti, M., Quarantelli, M., Sorrentino, G., Bernard C., & Jirsa, V. (2023). Supporting information for "Dynamical interactions reconfigure the gradient of cortical timescales. *Network Neuroscience, 7*(1), 73–85. https://doi.org/10.1162/netn_a_00270

Supplementary Material

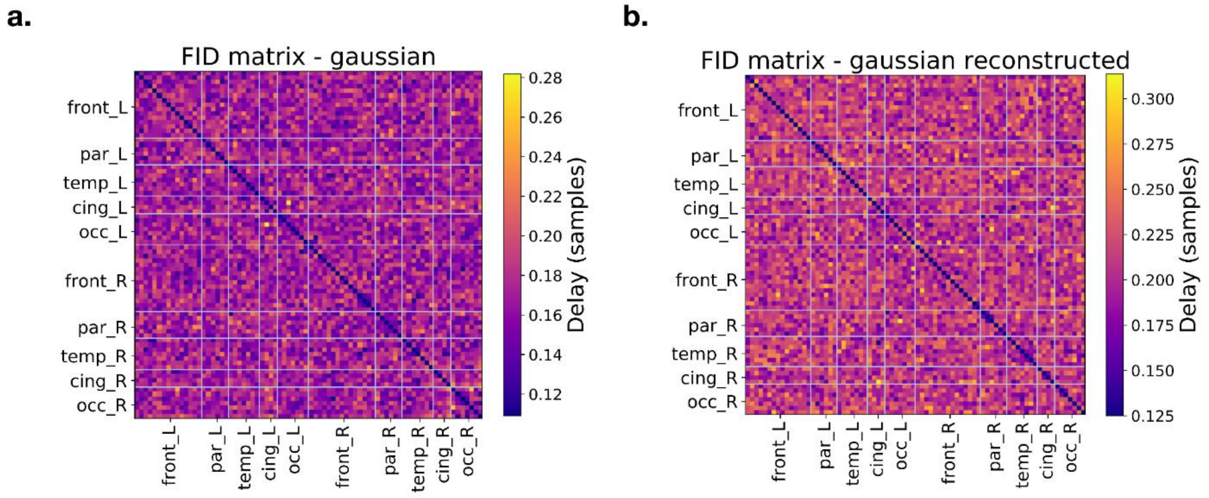

Fig. S1: Leakage analysis pipeline. a. FID matrix computed from Gaussian surrogates. The decay times were estimated from random surrogates analogously to the procedure used with the original data. Hence, rows and columns represent brain regions, while matrix entries are the corresponding decay minima. b. Starting from the Gaussian surrogates to the left, the surrogates were out projected, for each subject, using the corresponding mixing matrix, as to obtain new sensor-level surrogates. White noise, correlated as 1/distance between sensors, was added to the sensor-level surrogate with a SNR =12. The sensor-level surrogates were then inverted and new source-level data were generated. The minima of the decay of the AMI were then recomputed on the newly obtained source-level surrogates, to check that the spatial mixing of otherwise independent processes did not introduce the spatial patterns observed in the empirical data. The surrogate and empirical decay times did not show a statistically significant correlation (r=-0.01, p=0.43).

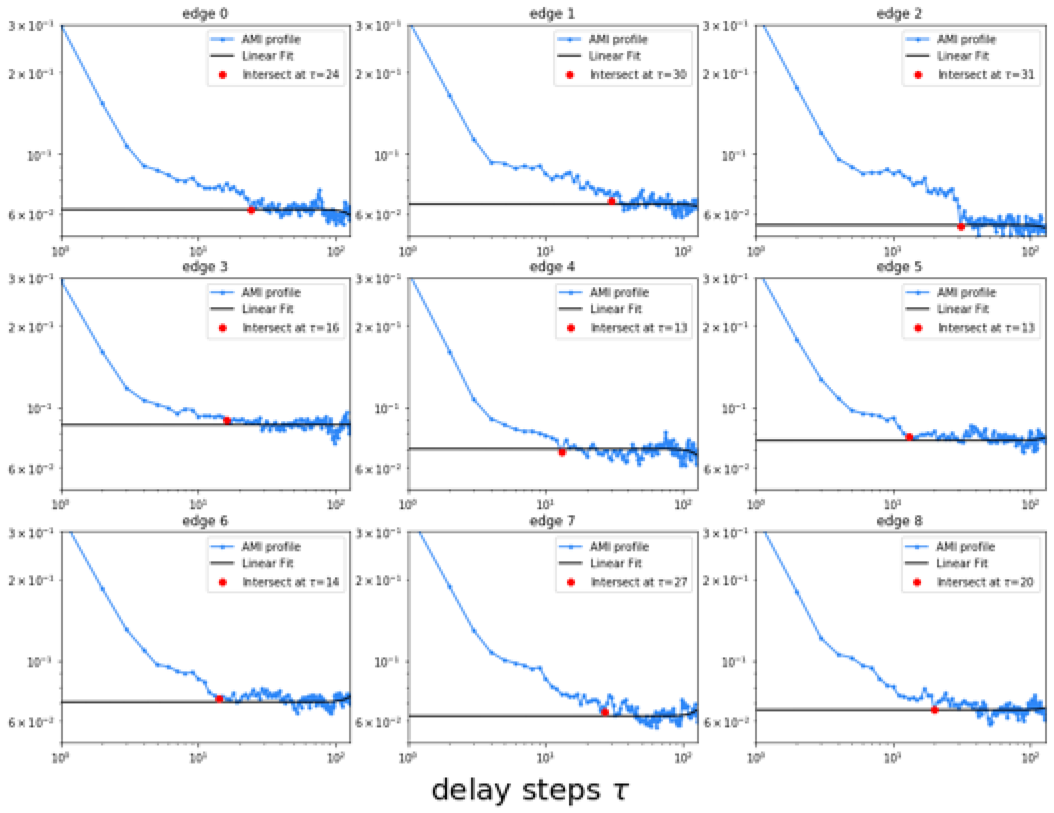

Fig. S2: Examples of the AMI decays of 9 randomly selected edges in a single trial (i.e., form the same 10 seconds-long epochs from a randomly selected subject) (log-log scale). In order to estimate the time after which the co-activation time series and its delayed version become maximally independent, we fit the last 80 points of the AMI profile (blue) to a straight line (black) and we find the first point (red) where the difference between the AMI decay and the fitted line is below a threshold. The results reported in the main text refer to the threshold set to 1 standard deviation from the AMI tail.

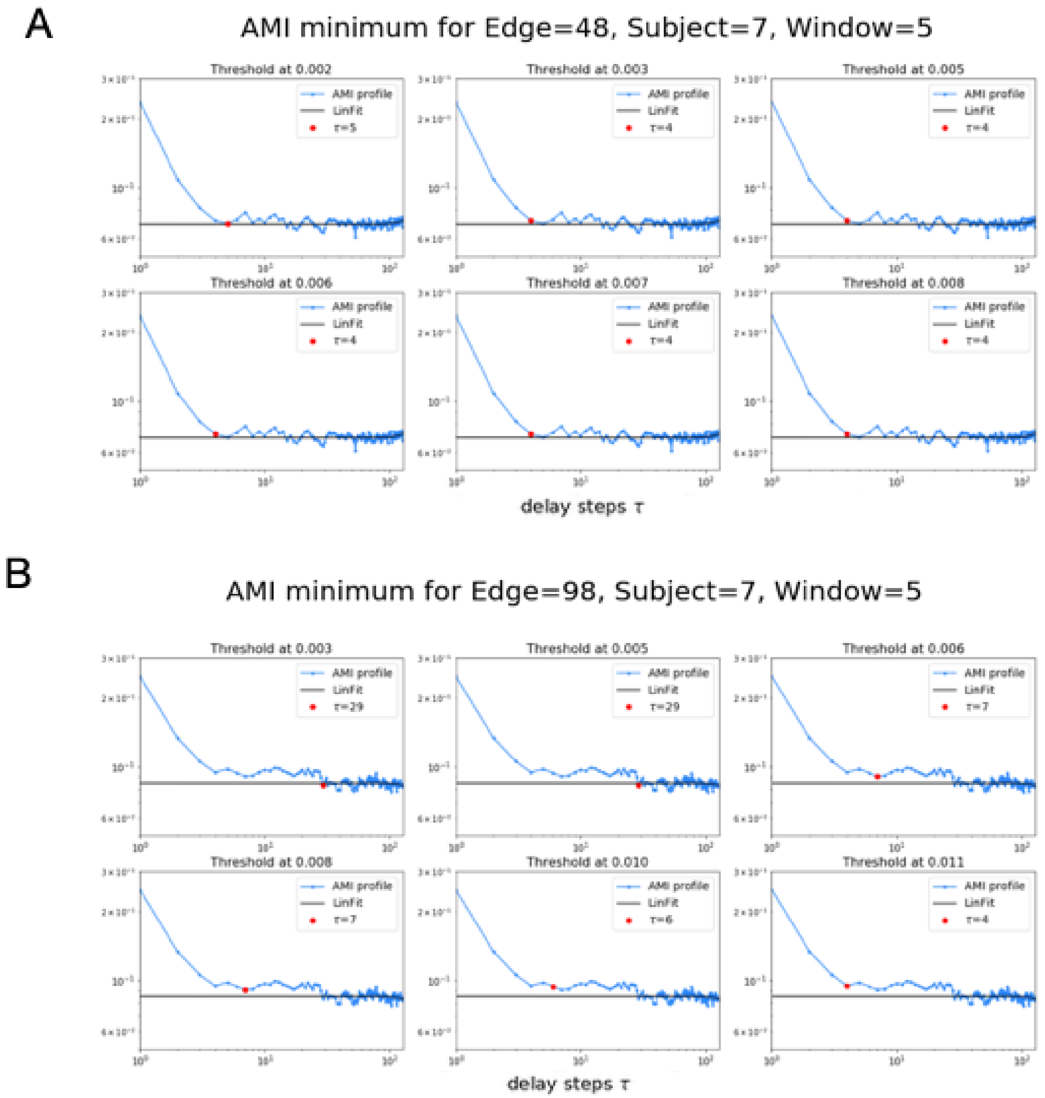

Fig. S3: The blue lines show the AMI decay profile for a single epoch. The red dots represent the estimate of the decay-times. The decay-time is measured as the first time that the AMI profile approaches the null-line (fitted to the tail of the AMI distribution, in black). The distance from the null-line, considered as a threshold, is defined as multiples of the standard deviation ([1, 1.5, 2., 2.5, 3., 3.5]) of the AMI tail. The six panels show the corresponding estimate of the decay time as the threshold is increased, from top-left to bottom right. (A) An example is drawn from a group of edges where the AMI decay profile approaches the minimum directly. The corresponding estimate of the decay time is stable across thresholds. (B) An example is drawn from a subset of edges where the decay profile does not approach the minimum directly. In such cases, the AMI decay profile shows a fat tail before reaching a stable minimum. Since we are interested in the

time required for the information to be completely extinguished in an edge, we considered the smallest threshold for the estimation of all the minima. This provides a stable estimate of the minimum over all the observed decay profiles.

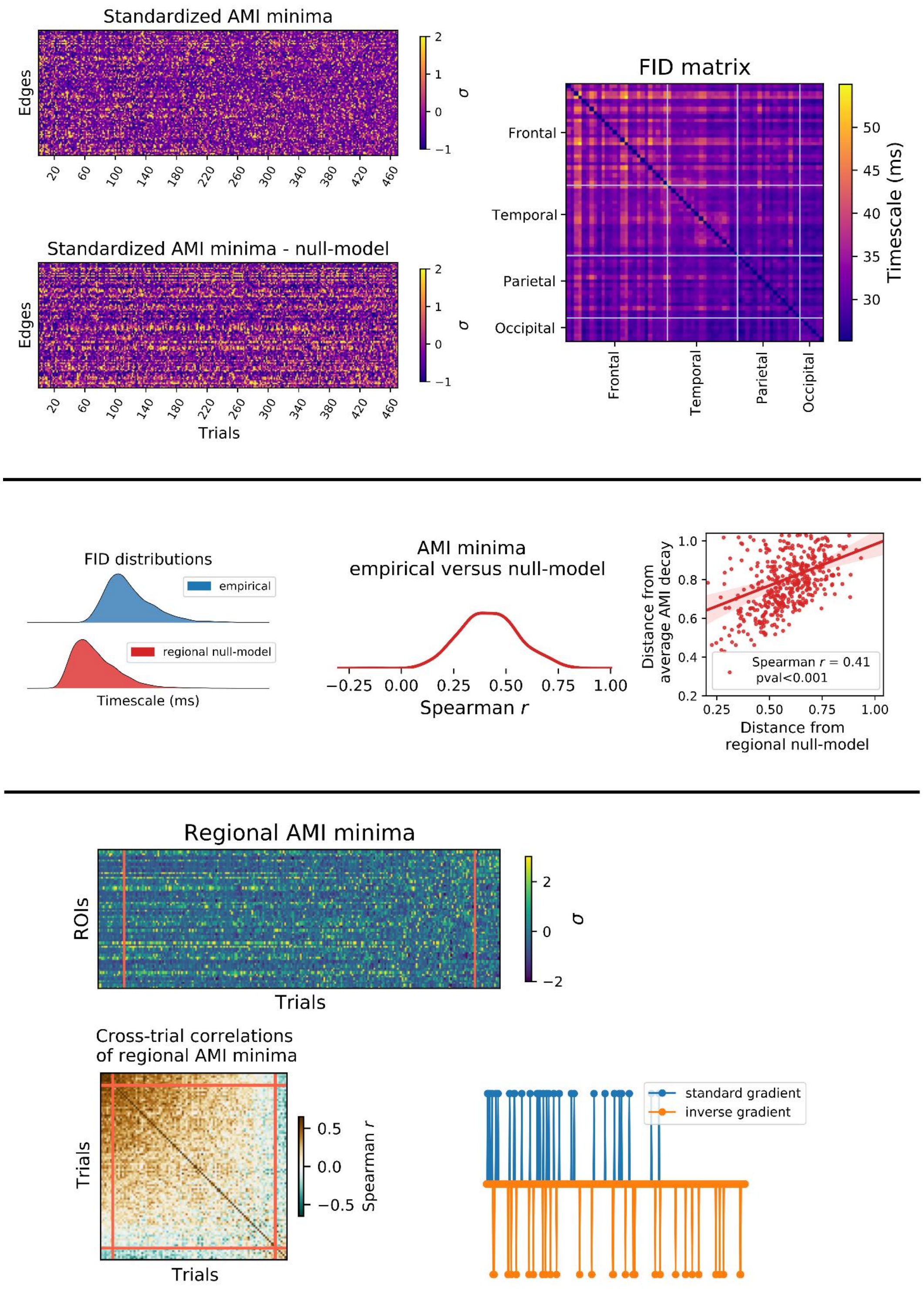

Fig. S4: The results of the paper (Fig.1,2,3) are reproduced on the DKT atlas.

Detailed analysis of the average edge topography

The SSN and LSN subnetworks are topographically distinct, as shown in Fig.2.a. Edges with short information storage are often incident upon occipital regions, although not uniformly. In fact, it appears that the cunei and the occipital gyri are specifically involved in the SSN. These regions roughly correspond to Brodmann areas 17, 18 and 19, and are known to be hierarchically related to the extraction of abstract shapes from the visual input. The cuneus directly receives inputs from the retina, that are subsequently processed up to the superior occipital gyrus, in the visual associative cortex, which is responsible of the perception of the properties of objects and plays a role, for instance, in face and object recognition (*1*, *2*). The occipital regions are connected bilaterally to the inferior parietal gyrus (e.g. the angular and supramarginal gyri as well as the inferior parietal lobe), the posterior temporal lobes and frontal/prefrontal cortex. The posterior part of the parietal lobe has been included in the “dorsal stream” of the visual system, which is mainly concerned with the analysis of spatial relationships of objects, and the information about the position of the body in space (*3*). With regard to the temporal lobe, the posterior part appears to be selectively involved in the SSN. From a structural standpoint, the inferior-longitudinal fasciculus (ILF), as well as an “indirect” stream of U-bundle fibers, connects the occipital and temporal areas (*4*). Functionally, these regions are related to either the high-level analysis of images, such as the inferior temporal lobe, whose lesion compromises the ability to recognize facial expressions, or to the perception of- and attendance to auditory stimuli, as in the case of the superior temporal lobe (*5*). Finally, in the frontal lobes, the SSN appears to involve the ventrolateral and dorsolateral prefrontal cortices, which participate to higher functions such as allocation of attention, and are typically considered the end of the ventral and dorsal streams, respectively (*6*, *7*). In short, all the regions that are known to be relevant in the process of sensory stimuli emerge as a temporally

homogeneous fast network carrying information from visual and acoustic areas, through layers of abstraction, up to areas related to conscious perception.

The opposite network, to which we refer to as the long storage network (LSN), clusters topographically in frontal and temporal regions. The regions involved in the LSN appear to be linked anatomically by the uncinate fasciculus. In fact, the LSN mainly connects the orbitofrontal cortex within the frontal cortex to the temporal poles in the temporal lobes. Patients who underwent brain surgery involving the removal of the uncinate fasciculus showed impairment of verbal memory, naming of objects, verbal fluency, and name anomia. These symptoms did not appear in patients whose surgery did not encompass the removal of the connections between the orbitofrontal cortex and the temporal lobes (*8*). This evidence might highlight the functional significance of the LSN for the integration of information across these anatomically linked regions. Furthermore, the semantic variant of primary progressive aphasia and herpes encephalitis, both diseases that induce a damage to the temporal poles, cause disruptions in categorical discrimination, word comprehension and naming (*9*). Recently, Warren et al. (*10*), using fMRI showed that, during narrative speech comprehension, the left anterior basal temporal cortex shows high correlation with the left anterior basal frontal cortex, the left anterior inferior temporal gyrus as well as with the corresponding homotopic temporal cortex contralaterally. From a neurophysiological perspective, the N400 response is an event-related potential (ERP) evoked ~400 ms after (potentially) meaningful material is presented. Using MEG, the N400 was localized in the superior temporal sulcus (*11*), and intracranial recordings showed it originates in the anteroventral temporal lobe. Furthermore, the SSN entails more long-range inter-hemispheric connections as compared to the LSN, which might suggest that inter-hemispheric coordination is specifically achieved via

fast interactions (perhaps specifically relying on fast, white-matter bundles such as the corpus callosum).

1. L. A. Renier, I. Anurova, A. G. De Volder, S. Carlson, J. VanMeter, J. P. Rauschecker, Preserved functional specialization for spatial processing in the middle occipital gyrus of the early blind. *Neuron*. **68**, 138–148 (2010).
2. E. Freud, J. C. Culham, D. C. Plaut, M. Behrmann, The large-scale organization of shape processing in the ventral and dorsal pathways. *eLife*. **6** (2017), doi:10.7554/eLife.27576.
3. M. A. Goodale, A. D. Milner, Separate visual pathways for perception and action. *Trends in Neurosciences*. **15**, 20–25 (1992).
4. G. Herbet, I. Zemmoura, H. Duffau, Functional Anatomy of the Inferior Longitudinal Fasciculus: From Historical Reports to Current Hypotheses. *Frontiers in Neuroanatomy*. **12**, 77 (2018).
5. M. Vander Ghinst, M. Bourguignon, M. Op de Beeck, V. Wens, B. Marty, S. Hassid, G. Choufani, V. Jousmäki, R. Hari, P. Van Bogaert, S. Goldman, X. De Tiège, Left superior temporal gyrus is coupled to attended speech in a cocktail-party auditory scene. *Journal of Neuroscience*. **36**, 1596–1606 (2016).
6. P. S. Goldman, H. E. Rosvold, Localization of function within the dorsolateral prefrontal cortex of the rhesus monkey. *Experimental Neurology*. **27**, 291–304 (1970).
7. F. de Pasquale, S. Della Penna, A. Z. Snyder, L. Marzetti, V. Pizzella, G. L. Romani, M. Corbetta, A Cortical Core for Dynamic Integration of Functional Networks in the Resting Human Brain. *Neuron*. **74**, 753–764 (2012).

8. C. Papagno, C. Miracapillo, A. Casarotti, L. J. Romero Lauro, A. Castellano, A. Falini, G. Casaceli, E. Fava, L. Bello, What is the role of the uncinate fasciculus? Surgical removal and proper name retrieval. *Brain*. **134**, 405–414 (2011).

9. U. Noppeney, K. Patterson, L. K. Tyler, H. Moss, E. A. Stamatakis, P. Bright, C. Mummery, C. J. Price, Temporal lobe lesions and semantic impairment: A comparison of herpes simplex virus encephalitis and semantic dementia. *Brain*. **130**, 1138–1147 (2007).

10. J. E. Warren, J. T. Crinion, M. A. Lambon Ralph, R. J. S. Wise, Anterior temporal lobe connectivity correlates with functional outcome after aphasic stroke. *Brain*. **132**, 3428–3442 (2009).

11. K. Marinkovic, R. P. Dhond, A. M. Dale, M. Glessner, V. Carr, E. Halgren, Spatiotemporal Dynamics of Modality-Specific and Supramodal Word Processing The importance of the left anterior temporal cortex for. *Neuron*. **38**, 487–497 (2003).
